# Morphological and Phylogenetic Characterisation of *Prorocentrum spinulentum*, sp. nov. (Prorocentrales, Dinophyceae), a Small Spiny Species from the North Atlantic

**DOI:** 10.3390/microorganisms11020271

**Published:** 2023-01-20

**Authors:** Urban Tillmann, Marc Gottschling, Stephan Wietkamp, Mona Hoppenrath

**Affiliations:** 1 Alfred Wegener Institute for Polar and Marine Research, Am Handelshafen 12, 27570 Bremerhaven, Germany; 2 Department Biologie—Systematik, Biodiversität & Evolution der Pflanzen, GeoBio-Center, Ludwig-Maximilians-Universität München, Menzinger Str. 67, 80638 München, Germany; 3 German Centre for Marine Biodiversity Research (DZMB), Senckenberg am Meer, Südstrand 44, 26382 Wilhelmshaven, Germany

**Keywords:** dinoflagellate, morphology, periflagellar area, phylogeny, plankton, protist, taxonomy

## Abstract

*Prorocentrum* comprises dinophytes with several unique traits, including the presence of two large thecal plates and apical insertion of flagella. Species delimitation for many small and similar planktonic species is challenging, as SEM analyses and DNA sequence information of type material are rarely available. Based on a strain from the North Atlantic *Prorocentrum spinulentum,* sp. nov. is described here. Cells were small (9.0–12.8 µm long, 8.5–11.9 µm deep), oval to almost round in lateral view and moderately compressed. The ovoid nucleus was in median or slightly sub-median position on the cells ventral side. The plate surface appeared spiny in light microscopy with thecal pores visible in empty thecae. Electron microscopy revealed plates densely covered by relatively long spines and two size classes of thecal pores. The periflagellar area consisted of 8 platelets, and there was a prominent wing (ca. 1 µm wide and long) on platelet 1. The new species is distinct in DNA trees and embedded in the *Prorocentrum shikokuense* species group. It differs from the protologues of other small species of *Prorocentrum* by the unique combination of cell size and shape, the presence of long spines on the thecal plate surface and scattered thecal pores. The thorough morphological description of this species, representing a previously uncharacterised lineage within *Prorocentrum,* increases and improves our knowledge of the diversity within this important group of planktonic organisms.

## 1. Introduction

Prorocentrales (Dinophyceae) have a peculiar morphology characterised by the presence of two major large thecal plates with a distinct sagittal suture, the lack of cingulum and sulcus and an apical flagella insertion (desmokont flagellation). Both flagella arise from one pore in the periflagellar area, which consists of tiny platelets and an additional accessory pore [1,2]. *Prorocentrum* Ehrenberg is diverse and comprises predominantly marine species with a worldwide distribution [1,2]. Identification of small (<20 µm), planktonic species of *Prorocentrum* is challenging [3]. They are inconspicuous, and differentiating traits, such as surface ornamentation of thecal plates, number and distribution of thecal pores and the presence/absence of apical projections, are difficult to observe unambiguously in light microscopy (LM). Moreover, most small species of *Prorocentrum* have been described a long time ago, and thus, detailed information on morphology or DNA sequence data linked to the types, or other original material, are lacking [3].

A good example of taxonomic confusion and ambiguity in *Prorocentrum* is a group, which contains sequences in molecular phylogenetics assigned to a variety of taxa such as *Prorocentrum dentatum* F.Stein, *Prorocentrum donghaiense* D.D.Lu, *Prorocentrum obtusidens* J.Schiller and *Prorocentrum shikokuense* Hada. Identity and synonymy of these taxa is debated [4,5], but ITS sequence data indicate that all may represent the same species. We agree with Gómez et al. [5] that the few morphological documentations of such strains conform with *P. shikokuense* (=*P. donghaiense*). In contrast, both *P. dentatum* (having a very characteristic shape) and *P. obtusidens* (being much larger) are morphologically different, and reliable sequence data for these taxa have not yet been obtained. We thus here refer to this group as the *P. shikokuense* species group.

Different from the old names of *Prorocentrum*, *Prorocentrum nux* Puigserver & Zingone has been described using scanning electron microscopy (SEM) [6] but even for this species, the only sequence data available in GenBank are from strain RCC303 (without accompanying morphological documentation) and not from the authentic strain pronap1, from which the type material has been prepared. *Prorocentrum ponticum* Krakhmalny & Terenko (*nom. corr.*: ICN Art. 23.5) is also documented using SEM, but no sequence data linked to type material have been gained [7]. In turn, a new clade of *Prorocentrum* has been identified based on ribosomal RNA (rRNA) sequence data referring to small cells identified as *Prorocentrum* cf. *balticum* (Lohmann) A.R.Loeblich, but their morphology is illusive at present [8].

The description of the new small, planktonic species *Prorocentrum pervagatum* Tillmann, Hoppenrath & Gottschling is based on multiple cultured strains from geographically distant localities and by combining high resolution LM and SEM with DNA sequence information [3]. Only a short time later, another small species, *P. thermophilum* F.Gómez, Tangcheng Li, Hu.Zhang & Senjie Lin, was described, together with other strains of *P. pervagatum* (=*P. criophilum* Gourvil & Gutiérrez-Rodríguez, syn. nov.) [9]. Such studies, combining molecular and morphological approaches, are the prerequisite for a solid taxonomic basis, facilitating a comprehensive comparison and reliable delimitation of other small species of *Prorocentrum*. Based on a strain isolated from the Celtic Sea (North Atlantic) in 2018, we provide here a next step in the characterisation of another small and new species of *Prorocentrum* using an integrative taxonomy approach.

## 2. Materials and Methods

### 2.1. Sampling, Cell Isolation, Cultivation

A single strain of *Prorocentrum* (1-D3) was established from a surface water sample (salinity: 34.9; surface temperature: 19.4 °C) collected at the Celtic Sea (51°1.31′ N; 9°4.21′ W) during a cruise aboard the research vessel *FS. Heincke* in 2018. Single cells were isolated by micropipetting under a stereomicroscope (M5A; Wild, Heerbrugg, Switzerland) and transferred into individual wells of 96-well tissue culture plates (TPP, Trasadingen, Switzerland), each containing 250 μL of K-medium [10] prepared from 0.2 μm sterile-filtered North Sea water diluted 1:10 with filtered seawater from the sampling locality. The original K-medium receipt was slightly modified by replacing the organic phosphorous source by 3.62 µM Na_2_HPO_4_. Plates were incubated at 15 °C under dim light (30 μmol photons m^−2^ s^−1^) in a controlled environment growth chamber (MIR 252; Sanyo Biomedical, Wood Dale, USA–IL). After 3–4 weeks, the strain was inoculated for batch culture in a 65 mL polystyrene cell culture flask. Growth medium was enriched with nutrients corresponding to 50% of K-medium.

For DNA extraction, cells were collected by centrifugation (5810R; Eppendorf, Hamburg, Germany) in 50 mL centrifugation tubes at 3220× *g* for 10 min. Cell pellets were transferred to 1 mL microtubes, then again centrifuged (5415; Eppendorf) at 16,000× *g* for 5 min and stored frozen (−20 °C) for subsequent DNA extraction. In addition, the strain was grown and harvested as described above for lipophilic toxin analysis and stored at −20 °C until use. For each harvest, cell density was determined by settling Lugol-fixed samples and counting >400 cells under an inverted microscope.

### 2.2. Microscopy

Observation of living or fixed cells (formaldehyde: 1% final concentration, or neutral Lugol-fixed: 1% final concentration) was carried out using an inverted microscope (Axiovert 200M; Zeiss, Jena, Germany) and a compound microscope (Axioskop 2; Zeiss), both equipped with epifluorescence and differential interference contrast optics. Light microscopic examination of thecal plates was performed using epifluorescence microscopy of cells stained with calcofluor white [11]. The shape and location of the nucleus was determined after staining of formalin-fixed cells with 4′-6-diamidino-2-phenylindole (DAPI, 0.1 µg mL^−1^ final concentration) for 10 min. Images were taken either with a digital camera (Axiocam MRc5; Zeiss), or videos were recorded using a digital camera (Gryphax; Jenoptik, Jena, Germany) at full-HD resolution. Single frames were then extracted using Corel Video Studio software (Version X8; Corel, Ottawa, ON, Canada). Cell length and depth of freshly fixed cells (neutral Lugol) from dense but healthy and growing strains (based on stereomicroscopic inspection of living material) during late exponential phase were measured at microscopic magnification of 1000× using the compound microscope and the Axiovision software (Zeiss).

For SEM, cells were collected by centrifugation of 15 mL culture (5810R; Eppendorf) at 3220× *g* for 10 min. The supernatant was removed and the cell pellet re-suspended in 60% ethanol prepared in a 2 mL microtube with seawater (final salinity ca 13) at 4 °C for 1 h in order to strip off the outer cell membrane. Cells were further collected by centrifugation (5415R, Eppendorf) at 16,000× *g* for 5 min and re-suspended and fixed in a 60:40 mixture of deionised water and seawater (final salinity ca. 13) with the addition of formaldehyde (1% final concentration) and stored at 4 °C for 3 h. Cells were collected on polycarbonate filters (Millipore Merck; Darmstadt, Germany; 25 mm Ø, 3 µm pore-size) in a filter funnel, in which all subsequent washing and dehydration steps were carried out. A total of eight washing steps (2 mL MilliQ-deionised water each) were followed by a dehydration series in ethanol (30%, 50%, 70%, 80%, 95%, 100%; 10 min each). Filters were dried with hexamethyldisilazane (HMDS), first in 1:1 HMDS:EtOH, followed by twice 100% HMDS and then stored in a desiccator under gentle vacuum. Finally, filters were mounted on stubs, sputter coated (Emscope SC500; Ashford, UK) with gold-palladium and viewed at 10 kV under a SEM (FEI Quanta FEG 200; Eindhoven, The Netherlands). Micrographs were presented on a black background using Photoshop 6.0 (Adobe Systems; San Jose, CA, USA).

### 2.3. DNA Sequencing

Genomic DNA was extracted from harvested fresh material using the NucleoSpin Plant II Kit (Macherey-Nagel; Düren, Germany) according to the manufacturers’ instructions. The DNA extract was stored at −20 °C until further processing. Amplification and Sanger-sequencing was performed for various regions of the rRNA genes, including the 18S/small subunit (SSU), the Internal Transcribed Spacer region (ITS1, 5.8S rRNA, ITS2) and the D1/D2 region of 28S/large subunit (LSU) using the following primer sets: 1F (5′-AAC CTG GTT GAT CCT GCC AGT-3′) and 1528R (5′-TGA TCC TTC TGC AGG TTC ACC TAC-3′) for SSU [12]; ITSa (5′-CCA AGC TTC TAG ATC GTA ACA AGG (ACT)TC CGT AGG T-3′) and ITSb (5′-CCT GCA GTC GAC A(GT)A TGC TTA A(AG)T TCA GC(AG) GG-3′) for ITS [13]; DirF (5′-ACC CGC TGA ATT TAA GCA TA-3′) and D2C (5′-CCT TGG TCC GTG TTT CAA GA-3′) for LSU [14].

Each PCR reaction contained 16.3 μL of ultra-pure H_2_O, 2.0 μL of HotMaster Taq buffer (5Prime; Hamburg, Germany), 0.2 μL of each primer (10 μM), 0.2 μL of dNTPs (10 μM), 0.1 μL of Taq Polymerase (Quantabio; Beverly, MA, USA) and 1.0 μL of extracted DNA template (10 ng μL^−1^) to a final reaction volume of 20 μL. PCRs were conducted in a Nexus Gradient Mastercycler (Eppendorf) with conditions previously described [15], and PCR amplicons were checked by electrophoresis on a 1% agarose gel (in TE buffer, 70 mV, 30 min) to verify the expected lengths. Amplicons were purified following the instructions of the NucleoSpin Gel and PCR clean-up kit (Macherey-Nagel) and were sequenced directly in both directions on an ABI PRISM 3730XL (Applied Biosystems by Thermo Fisher Scientific, Waltham, MA, USA) as previously described [16]. Raw sequence data were processed using the CLC Genomics Workbench 12 (Qiagen; Hilden, Germany).

### 2.4. Molecular Phylogeny

A systematically representative set of prorocentralean and related accessions was compiled from known reference trees, similarly to a previous approach [3]. The taxon sample was enriched by all those accessions showing ultimate similarity to the sequences gained from strain 1-D3, as inferred from BLAST searches [17]. Voucher information is provided in Table S1, which also includes GenBank accessions of *Prorocentrum spinulentum* (OQ220500, OQ220501), and outgroup details comprising Dinophysales and Gymnodiniales. For alignment, separate matrices of the rRNA operon (i.e., SSU, ITS, LSU) were constructed, aligned using MAFFT v6.502a [18] and then concatenated. The aligned matrices are available as a file named ‘spinulentum.nexus’ upon request. Phylogenetic analyses were carried out using maximum likelihood (ML) and Bayesian approaches as described previously [19].

### 2.5. Terminology

Terminology of cell orientation, designation of thecal plates and platelets and ornamentation follows Hoppenrath et al. [2] and includes some additions and modifications as discussed by Tillmann et al. [20].

## 3. Results

### 3.1. Formal Description

**Figure 1:**
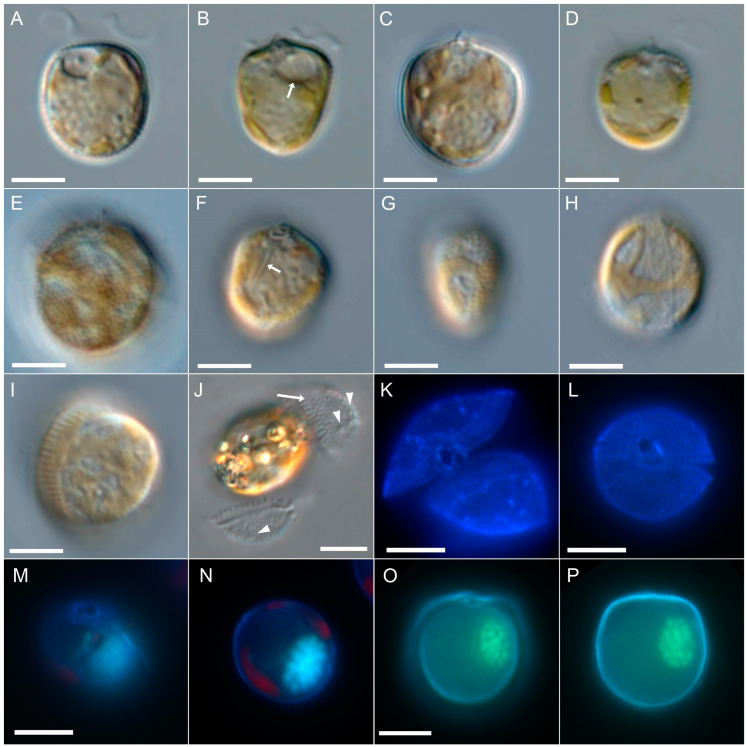
*Prorocentrum spinulentum*, strain 1-D3. LM of living cells (**A**–**J**) or formaldehyde-fixed cells (**K**–**P**). Different cells in lateral view (**A**–**F**), or in dorsal/ventral view (**G**–**I**), note the pusule (white arrow in (**B**)), long trichocysts rods (white arrow in (**F**)), the spiny plate surface (**E**) and the broadly striated sagittal suture (**H**,**I**); (**J**) thecal plates detached from the cell, note the striated suture (white arrow) and the visibility of thecal pores (arrowheads); (**K**,**L**) cells stained with calcofluor white and viewed with epifluorescence and UV light excitation in apical view; (**M**–**P**) cells stained simultaneously with calcofluor white and DAPI to indicate the ventral location of the ovoid nucleus; (**M**,**N**) the same, DAPI stained cell viewed in different focal planes; (**O**,**P**) the same DAPI stained cell viewed in different focal planes; scale bars = 5 µm.

**Figure 2:**
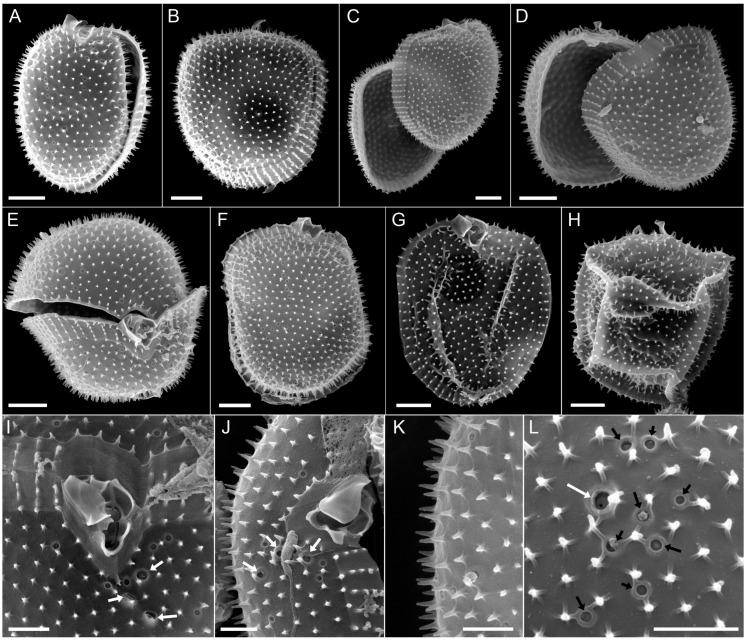
*Prorocentrum spinulentum,* strain 1-D3. SEM of different thecae; (**A**,**B**) cells in right-lateral view; (**C**,**D**) cells with left and right lateral plates slightly detached; (**E**) cell in apical view; (**F**,**G**) right thecal plates to indicate position of thecal pores; (**H**) cell in left-lateral view to indicate the arrangement of apical projections; (**I**,**J**) apical views of the periflagellar area, note the arrangement of three large pores (white arrows); (**K**,**L**) surface structure of thecal plates, note the three-dimensional shape of the spines, the structures at the spine base and the presence of small (black arrows) and large (white arrow) thecal pores; scale bars = 2 µm (**A**–**H**) or 1 µm (**I**–**L**).

**Figure 3:**
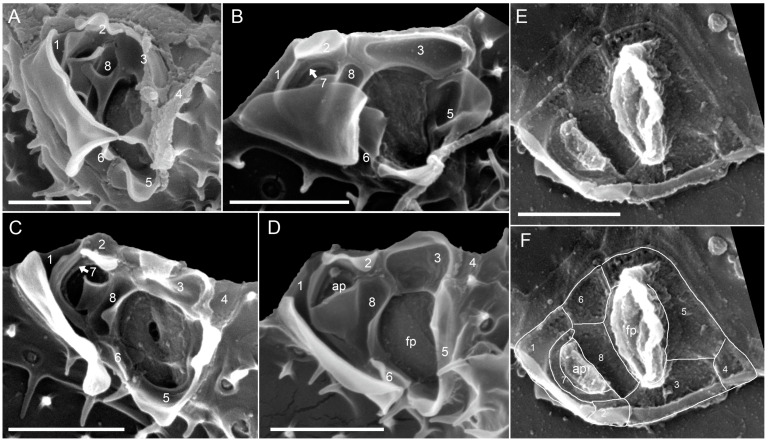
*Prorocentrum spinulentum*, strain 1-D3: (**A**–**E**) detailed apical views of the periflagellar area in external (**A**–**D**) or internal view (**E**,**F**); (**F**) the same view as (**E**), but with plate sutures accentuated by lines; numbers indicate nominations of the periflagellar platelets, fp = flagellar pore; ap = accessory pore; scale bars = 1 µm.

*Prorocentrum spinulentum* Tillmann, Gottschling & Hoppenrath, sp. nov. (Figure 1, Figure 2 and Figure 3).

Description: Small, photosynthetic, thecate, prorocentroid Dinophyceae with desmokont flagellation; cell shape irregularly oval to round in lateral view or rarely heart-shaped, 9.0–12.8 µm in length, 8.5–11.9 µm in depth, with a mean l/d ratio of 1.1. Cells slightly compressed laterally after division or almost globose in older cells with a broad and transversely striated intercalary band; two reticulate chloroplasts, an oval-shaped nucleus in ventral position and a pusule in apical position close to the flagellar pore. Apical projection visible in LM. Thecal plates covered by elongated spines, with thecal pores of two different size classes, in total about 30 pores per plate, scattered mainly towards the plate margin or arranged in two short lines close to the thecal centre. A cluster of three to four large pores located on the right theca in apical ventral position. Periflagellar area composed of 8 platelets, accessory and large flagellar pores, with projections present on platelets 1, 2, 3, 5, 6 and 8. An elongated conspicuous wing (width and height ca. 1 µm each) running all along the right margin of platelet 1 as the most prominent apical projection.

Holotype: SEM-stub prepared from clonal strain 1-D3 (designated CEDiT2023H159) deposited at the Senckenberg Research Institute and Natural History Museum, Centre of Excellence for Dinophyte Taxonomy, Germany.

Isotypes: Formalin-fixed sample prepared from clonal strain 1-D3 (designated CEDiT2023I160) deposited at the Senckenberg Research Institute and Natural History Museum, Centre of Excellence for Dinophyte Taxonomy, Germany.

Type locality: Northeast Atlantic, off southern Ireland (51°01.31′ N; 09°04.21′ W).

Habitat: Coastal water (plankton).

Strain establishment: The strain was sampled and isolated by U. Tillmann on 26 July 2018.

Etymology: The epithet (Lat. *spinulentus* = spinose) reflects the spinose surface of both lateral plates provided by rather long spines (compared to the small cell size).

PhycoBank registration: http://phycobank.org/103590.

### 3.2. Detailed Description

#### 3.2.1. Light Microscopy

Cells were asymmetrically oval to round or rarely heart-shaped in lateral view (Figure 1). Cell length ranged from 9.0 to 12.8 (mean 10.9 µm ± 0.8 µm, *n* = 50), and cell depth ranged from 8.5 to 11.9 (mean 10.3 µm ± 0.8 µm, *n* = 50), with a mean l/d ratio of 1.07. Low magnification observation of living and swimming/turning cells indicated a wide range of length/width ratio (Figure 1G,H) ranging from ca. 0.6 to 0.9, with widest cells dominating in dense cultures. Wide cells had a broad and transversely striated growth band visible in LM (Figure 1H,I). Both plates were covered by spines visible in LM (Figure 1E). Thecal pores were difficult to detect in living or fixed cells in brightfield or differential interference contrast microscopy, but were faintly visible on empty thecae (Figure 1J) and most easily seen in calcofluor-stained cells under UV excitation (Figure 1K,L). An apical projection was occasionally visible in LM (Figure 1B–E). Two yellow-orange, reticulate chloroplasts were arranged parietally adjacent to the thecal plates, and no pyrenoid was observed in LM. A small, round, hyaline, vacuole-like structure, the pusule, was occasionally visible in the anterior area close to the flagellar pore (Figure 1A,B). Long, rod-like structures, presumably trichocysts, were visible in LM and mainly arranged along the cell’s longitudinal axis (Figure 1F). The nucleus was oval in outline and located in the ventral area of the cell in median or submedian position (Figure 1M–P). One large flagellar pore and a distinctly smaller accessory pore in apical position could be adumbrated in LM (Figure 1F) and was clearly visible in calcofluor-stained cells under UV excitation (Figure 1K–M).

#### 3.2.2. Scanning Electron Microscopy

The two thecal plates were uniformly covered by spines (Figure 2), which ranged from 0.30–0.43 µm (Table 1) in length. At the base of each spine, there were three to five radial extensions (Figure 2L). Mean areal density of spines ranged from 4.2 to 5.0 µm^−2^ (Table 1). The transverse striation of the intercalary band consisted of rows of spines (Figure 2B–E). Details of the thecal pores could be revealed (Figure 2I–L). Larger pores were about 0.23 µm in diameter (Table 1) and bordered by a crater-formed rim at the outer plate surface (Figure 2I,L). Small pores were about 0.14 µm in diameter (Table 1) and were also bordered by a small elevated rim (Figure 2L). A cluster of two to three large pores accompanied by several small pores were characteristically visible on the right thecal plate in apical ventral position (Figure 2I,J). Other pores were scattered on both thecal plates with two distinct rows of pores close to the plate centre (Figure 2A–D,F). There were about 30 pores per plate (mean: 32 ± 6, range 20–51), only a few (ca. 3–5) of them were large (the exact number was difficult to determine).

The periflagellar area (Figure 3) was about 2.2 µm deep and 1.5 µm wide (Table 2) and located between both thecal plates in a broadly V-shaped indentation of the right plate (Figure 2E,I). There were a number of apical projections, which were dominated by a conspicuous wing (Figure 2A–J and Figure 3A–D) of about 1 µm each in width and length (Table 2). In the periflagellar area, eight platelets (1, 2, 3, 4, 5, 6, 7 and 8) surrounded a flagellar pore (fp) and an accessory pore (ap) (Figure 3). The fp was of irregular oval shape and generally longer than wide (ca. 0.9 µm long, 0.6 µm wide, Table 2) and surrounded by platelets 3, 5, 6 and 8. The ap was smaller (ca. 0.6 µm long, 0.4 µm wide, Table 2) and surrounded by platelets 7 and 8. Platelet 2 was part of the upper rim of the ap but was separated by platelet 7 from the inner ring of the ap (Figure 3B). Both pores were internally closed by two lip-like structures (Figure 3C–F).

**Figure 4:**
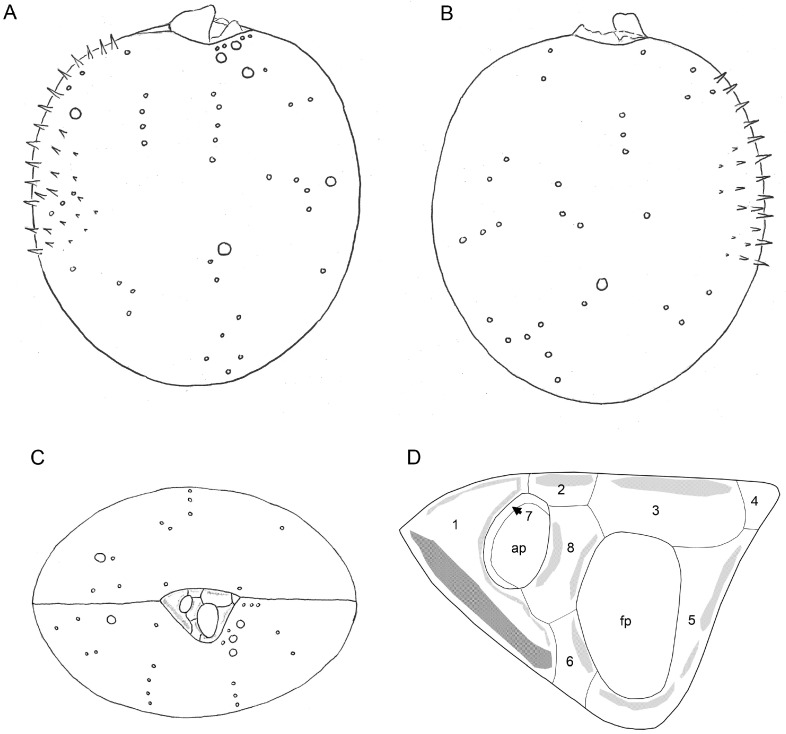
*Prorocentrum spinulentum*, schematic drawing of a representative pore pattern of the right (**A**) and the left theca (**B**), and of a cell in apical view (**C**). Density of thecal spines is partly indicated in (**A**,**B**); (**D**) schematic drawings of the periflagellar area; numbers indicate nominations of the periflagellar platelets; ap = accessory pore; fp = flagellar pore.

Platelet 1 in dorsal position carried the prominent wing, which bordered the right lateral side of platelet 1 (Figure 3A–D). Additional lists on platelet 1 provided the outline of the ap. Other apical projections were present on platelets 2, 3, 5, 6 and 8 (Figure 3A–D) and were variable in dimensions. Platelet 4 was small, triangular and plain and formed an acuminate ventral termination of the periflagellar area. From an internal view, both fp and ap had a tube-like appearance, and the plate sutures of the periflagellar platelets were revealed (Figure 3E,F). Schematic drawings of *P. spinulentum*, including of the periflagellar area, are compiled in Figure 4.

### 3.3. Molecular Phylogeny

**Figure 5:**
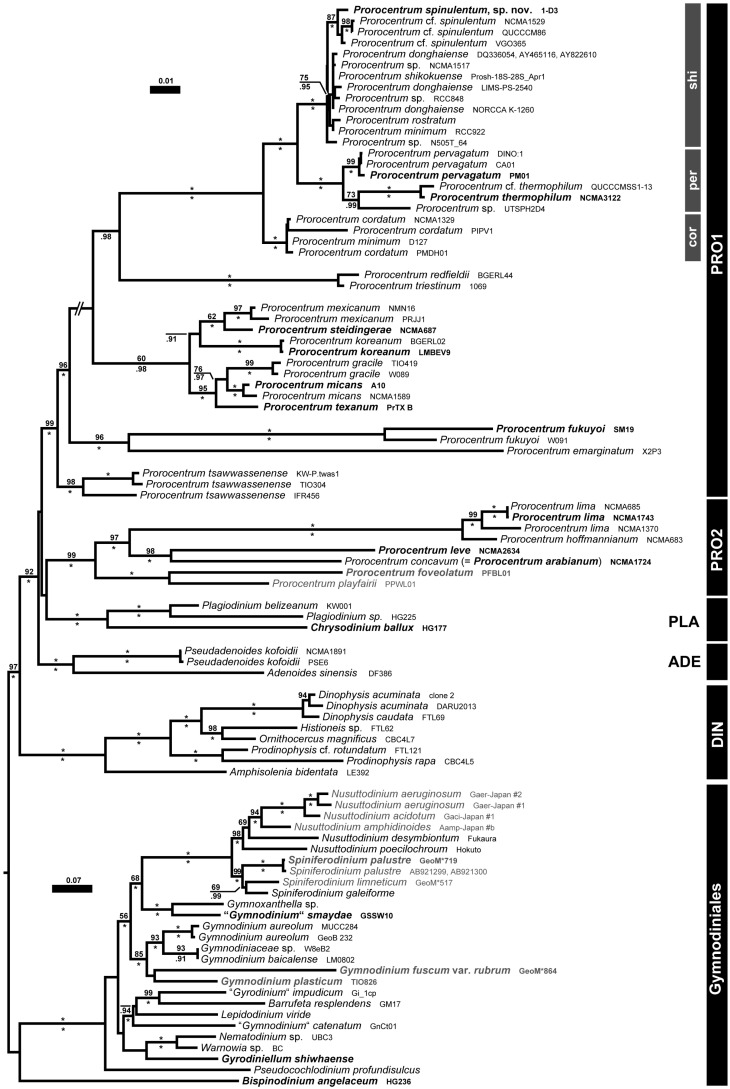
Molecular phylogenetics of prorocentralean dinophytes, including all accessions assignable to *P. spinulentum*. ML tree (−ln = 56,135.99), as inferred from an rRNA nucleotide alignment (1672 parsimony-informative sites) with strain number information. Accessions corresponding to type or at least reference material are indicated by bold type; freshwater accessions are shown in gray. Numbers on branches are ML bootstrap (above) and Bayesian support values (below) for the branches (asterisks indicate maximal support values; values under 50 and 0.90, respectively, are not shown). Clades are indicated (abbreviations: ADE, *Adenoides*; DIN, Dinophysales; GYM, Gymnodiniales; PLA, *Plagiodinium*; PRO, *Prorocentrum*, cor, *P. cordatum* species group; per, *P. pervagatum* species group; shi, *P. shikokuense* species group).

The SSU + ITS + LSU alignment was 1800 + 746 + 3491 bp long and composed of 293 + 532 + 847 parsimony-informative sites (28%, mean of 18.58 per terminal taxon) and 2731 distinct RAxML alignment patterns. Figure 5 shows the best-scoring ML tree (−ln = 56,135.99, being highly similar to the Bayesian tree), with many nodes having high if not maximal support. With respect to Dinophysales and Gymnodiniales, four lineages, including *Adenoides* Balech (100LBS, 1.00BPP), *Plagiodinium* M.A.Faust & Balech (100LBS, 1.00BPP) and *Prorocentrum*, formed a well-supported clade (92LBS, 1.00BPP). *Prorocentrum* was comprised of two clades, denominated here as PRO1, including the type species *Prorocentrum micans* Ehrenberg (99LBS, 1.00BPP), and PRO2 (99LBS, 1.00BPP).

The PRO1 clade consisted of five well-supported lineages, two of which comprised benthic species, such as *Prorocentrum tsawwassenense* Hoppenrath & B.S.Leander (98LBS, 1.00BPP) and *Prorocentrum fukuyoi* Sh.Murray & Nagahama (96LBS, 1.00BPP). The third lineage constituted the *P. micans* species complex (60LBS, 0.98BPP), whereas the fourth lineage included accessions of *Prorocentrum triestinum* J.Schiller and relatives (100LBS, 1.00BPP). The fifth lineage (100LBS, 1.00BPP) was segregated into three clades, including strains of *Prorocentrum cordatum* (Ostenf.) J.D.Dodge (100LBS, 1.00BPP), *P. pervagatum* (73LBS, 0.99BPP) and *P. shikokuense* (100LBS, 1.00BPP), with their respective relatives. The new species *P. spinulentum* was embedded in the clade of *P. shikokuense*, together with three other accessions (100LBS, 1.00BPP) showing low sequence divergence and leaving the remainder of the clade phylogenetically unresolved.

## 4. Discussion

The description of *Prorocentrum spinulentum* provides a scientific name for a small, already recognised and early branching subclade [3,9] of the *P. shikokuense* species group. Strains of this species group are described as having knob-like short spines on the thecal plate surface [4,21,22] and are thus different from *P. spinulentum* having long spines. Moreover, cells of *P. shikokuense* are larger and elongate [5] and thus cannot be confused with the smaller and in outline roundish *P. spinulentum*.

Two strains similar to *P. spinulentum* (i.e., CCMP1529 and RCC922) have been previously identified as *P. cordatum*, although the latter species is much larger. As inferred from the protologue of *P. cordatum* the cells have a surface ornamentation due to fine pores [23] but in SEM, rather long spines on the thecal plates are present [1], being very similar to *P. spinulentum*. Details of apical structures are not available from the original description of *P. cordatum*, but in SEM, a characteristic ‘double wing’ is present on platelet 1 (denoted as double-layered, curved apical collar) as well as spine-like projections (denoted as forked, single, and small teeth) on other periflagellar platelets [24,25,26]. In contrast, *P. spinulentum* has a characteristically different, wide and long wing on platelet 1.

There are 14 species of *Prorocentrum* (occasionally introduced under *Exuviaella* Cienkowski) of small size (<20 µm) with a roundish shape in outline that have already been compiled [3] (see Table 5 and Figure 7 therein). Among those, *P. spinulentum* differs from *Exuviaella equatorialis* Hasle (19 µm), *Prorocentrum antarcticum* (Hada) Balech (15–23 µm) and *Prorocentrum rotundatum* J.Schiller (16–21 µm) by size. Moreover, it differs in shape from *Prorocentrum cornutum* J.Schiller, which has a unique posterior horn. Detailed morphological features of all other small species of *Prorocentrum* [3] and the newly described *P. thermophilum*, are compiled and contrasted with *P. spinulentum* in Table 3. With the rather long thecal spines that *P. spinulentum* shares with *P. cordatum* only, it can be differentiated from species having a smooth surface (*P. nux*, *P. cordiforme* Bursa) or knob-like flat structures on the thecal plates (i.e., *P. pervagatum*, *P. ponticum*, *P. termophilum*; Table 3). Furthermore, *P. spinulentum* lacks a distinct apical spine and can therefore be differentiated from species with such a spine, including *P. cordiforme* (described with a short simple spine), *P. nanum* J.Schiller (described with a short spine), *P. spheroideum* J.Schiller (described with one solid spine) or *P. pomoideum* Bursa (described with one main apical tooth) (Table 3).

There are other small species of *Prorocentrum* without an apical spine and whose plate surface structure has not been described, and these are more difficult to differentiate from *P. spinulentum*. The distinct ovate outline of *Prorocentrum ovum* J.Schiller in lateral view differs from the broad oval to round outline shape of *P. spinulentum*. *Prorocentrum pusillum* J.Schiller shares with *P. spinulentum* the small size and lack of an apical spine, but it has strong lateral compression compared to the broader *P. spinulentum*. *Prorocentrum balticum* from the German Baltic Sea [27] is a frequently encountered small species [28,29,30] and has a very similar size compared to *P. spinulentum*. However, whether such determinations in fact represent the organism is challenging due to the gross similarity of the small species. The original description does not mention presence or absence of an apical spine or other obvious apical projections, and pores or other surface features of the thecal plates are not reported in the protologue as well. However, it can be argued that a spiny plate surface, as it is visible in LM for *P. spinulentum,* would have been noticed by Hans Lohmann and thus differentiates both species.

*Prorocentrum spinulentum* thus differs from all other similar species of *Prorocentrum* by its unique combination of cell size, shape and type of plate surface structures (i.e., spines). Moreover, *P. spinulentum* has a nucleus in median or sub-median position on the cell’s ventral side. Shape and/or position of the nucleus is rarely reported for the small species, but if available, the more subspherical or spherical nucleus (*P. pervagatum* [3], *P. thermophilum* [9], *P. cordatum* [36], *P. nux* [6]) has a posterior position. Other structural details outlined for *P. spinulentum* are the presence of two size classes of thecal (and presumably trichocyst) pores, which similarly have been reported for other species of the species complex, such as *P.* cf. *balticum*, *P. pervagatum* and *P. thermophilum* [3,8,9]. However, large pores of *P. spinulentum* (0.23 µm in diameter, Table 1) may be slightly smaller than large pores of *P. pervagatum* (0.26–0.34 µm [3]) or *P. thermophilum* (0.3 µm [9]). Strikingly, the few available SEM studies of related *P. shikokuense* report from small pores only (with a size of 0.15 µm corresponding to the small pores of *P. spinulentum*), which are irregularly distributed on the thecal plates, and explicitly deny size classes of thecal pores [4]. However, the plate surface structure of *P. spinulentum* hampers an unequivocal identification of large pores being rare (and that is why the quantification for *P. spinulentum* of thecal pores per plate did not differentiate small and large pores). More detailed, high-magnification SEM may uncover the presence or absence of large thecal pores in the *P. shikokuense* species group more reliably.

A number of small, in outline roundish species of *Prorocentrum*, namely *P. spinulentum* (this work, Figure 2I) and *P. pervagatum*, *P. thermophilum*, *P.* cf. *balticum* and *P. nux* [3,6,8,9], shares the presence of a distinct, short row of large pores in apical position on the right thecal plate. However, this trait has not yet been explicitly reported for the *P. shikokuense* species group or *P. cordatum*, but more detailed SEM observations of the periflagellar area are needed to better assess the phylogenetic importance of this trait. The periflagellar area of *P. spinulentum* is very similar in number (i.e., eight) and arrangement of the platelets to *P. cordatum* [38,39,40] and *P. pervagatum* [3]. However, *P. pervagatum* but not *P. spinulentum* has a distinct spine on platelet 6, and *P. spinulentum* but not *P. pervagatum* has a characteristic broad wing on platelet 1. A very similar arrangement of the periflagellar platelets is also shared between *P. shikokuense* ([4], as *P. obtusidens*) and *P. spinulentum*, and both species have a similar broad wing on platelet 1 as the dominant periflagellar structure. However, a subdivision of platelet 5 is present in *P. shikokuense* but absent in *P. spinulentum*. For *P. thermophilum*, knowledge of the periflagellar area is rather diffuse (“pores, surrounded by folded structures” [9]), and the number and arrangement of the periflagellar platelets remains unclear. The same is true for the preliminary and incomplete morphological description of *P.* cf. *balticum* [8]. More detailed SEM studies of more strains and species are needed to finally evaluate the suitability of such differences to distinguish the great diversity seen in the small species of *Prorocentrum*.

In conclusion, the recent descriptions of three new, small, planktonic species of *Prorocentrum*, namely *P. pervagatum*, *P. thermophilum* and *P. spinulentum* herein, provide a major step towards an unambiguous application of scientific names in *Prorocentrum* but with the clarification of many still unresolved and predominantly old names of *Prorocentrum*, there is a long way to go, preferably by using an epitypification approach based on material from the corresponding type localities [41,42].

## Figures and Tables

**Table 1:** Morphometry of thecal spines and pores in *Prorocentrum spinulentum* (strain 1-D3).

Spines		Pore Size	
Length (µm)	Density (µm^−2^)	Large (µm)	Small (µm)
Mean ± SD Min–Max	mean ± SD Min–Max	Mean ± SD Min–Max	Mean ± SD Min–Max
**0.36** ± 0.03 0.30–0.43 *n* = 18	**4.6** ± 0.2 4.2–5.0 *n* = 12	**0.23** ± 0.02 0.20–0.28 *n* = 13	**0.14** ± 0.01 0.11–0.16 *n* = 25

**Table 2:** Morphometry of the periflagellar area *Prorocentrum spinulentum* (strain 1-D3).

Periflagellar Area		Size Accessory Pore		Size Flagellar Pore		Size Wing on Platelet 1	
Depth (µm)	Width (µm)	Length (µm)	Width (µm)	Length (µm)	Width (µm)	Length	Height
Mean ± SD Min–Max	Mean ± SD Min–Max	Mean ± SD Min–Max	Mean ± SD Min–Max	Large	Small	Mean ± SD Min–Max	Mean ± SD Min–Max
**2.15** ± 0.17 1.78–2.37 *n* = 15	**1.48** ± 0.20 1.14–1.83 *n* = 15	**0.59** ± 0.03 0.54–0.63 *n* = 10	**0.38** ± 0.03 0.33–0.44 *n* = 10	**0.94** ± 0.11 0.74–1.12 *n* = 13	**0.60** ± 0.04 0.50–0.67 *n* = 12	**1.24** ± 0.13 0.93–1.49 *n* = 25	**1.09** ± 0.17 0.83–1.44 *n* = 20

**Table 3:** Comparison of small *Prorocentrum* species.

trait	*P. spinulentum*	*P. pervagatum*	*P. thermophilum*	*P. cordatum* ^1^	*P. balticum*	*P. sphaeroideum* ^2^	*P. nux*	*P. nanum*	*P. ovum*	*P. pusillum* ^3^	*P. ponticum*	*P* *. pomoideum* ^4^	*P. cordiforme* ^5^
shape in outline	round	round	almost round	obcordate emarginate heart-shaped	round, ovate	round	irregularily ovate	irregular, almost angular	ovate, anterior end square ^6^	ovate	orbicular to broadly elliptic	apple-shaped (often with posterior constriction)	heart-shaped
length [µm]	9.0–12.8	12–16	12–17	22–24 18	9–12	13	6.3–9	10–14	14	8–10	10.4–15.1	9–20	6–12
depth [µm]	8.5–11.9	12–16	10–15	18–20 15	9–12	13	5.3–10	10–14	10	6.5–7.5	9.6-11.5	11–16	5–9
compression	slightly compressed	young cells slightly compressed; round	no	compressed strongly compressed	no	not reported	no	strongly compressed	scarcely compressed	strongly compressed	slightly or strongly compressed	flattened	not reported
nucleus shape; position	ellipsoid; median, ventral	spherical to ellipsoid; posterior	more or less rotund; posterior	not reported not reported ^7^	not reported ^8^	not reported	round; posterior	not reported	not reported	not reported	shape not reported; posterior position	subspherical; posterior	not reported
apical spine	absent	1.3–1.7 µm, on platelet 6	absent	absent small tooth with minute wing	absent	a solid spine without wing	absent	short spine ^9^	absent	absent	absent	one main apical tooth without a wing	short simple spine ^10^
projections	high and wide wing on platelet 1	flat wings	spine-like prolongation	absent absent	not reported	not reported	absent	two irregular bumps bordering the flagellar pore	absent	absent	small projections	two more and smaller “spines”	additional smaller spine opposite to the main spine
surface	spines	knobs	knobs	with fine pores ^11^ densly perforated with subtle pores ^12^	not reported	not reported	smooth	not reported	not reported	with subtle pores ^13^	knobs	spines or smooth ^14^	smooth
spine/knob density [µm^−2^]	4.2–5.0	7.0-10.5	5.0–7.0	not reported not reported	not reported	not reported	not applicable	not reported	not reported	not reported	9 ^15^	not reported	not applicable
periflagellar plateletts	8	8	not determined	not determined not determined ^17^	not determined	not determined	7	not determined	not determined	not determined	not determined	not determined	not determined
pores	present	present	present	not reported not reported	not reported	present	present	present	present	present	one line of fine pores positioned in periphery of each thecal plate	numerous small pores	absent ^16^
pore size	large (0.23 µm), small (0.14 µm)	large (0.3 µm), small (0.16 µm)	large (0.3 µm), small (0.15 µm)	not reported not reported	not reported	large	large, small	large	large	subtle	fine pores	small	absent
pore number	ca. 30 per plate	ca. 20–30 per plate	ca. 10–15 per plate	not reported not reported	not reported	few	few	scarce	few	not specified or drawn	not specified	numerous	absent
pore position	scattered over plates	scattered on plate margins	scattered on plate margins	not reported not reported	not reported	all over theca	adjacent to plate margins	scattered all over theca	scatted all over theca	not specified	arranged in one line in periphery of each thecal plate	scattered all over theca	absent
type locality	Celtic Sea	Labrador Sea	Gulf of Mexixo, Sarasota, Florida	Caspian Sea Golfe de Lion, France	Baltic Sea (Kiel, Germany)	Adriatic Sea	Tyrrhenian Sea, off Naples	Adriatic Sea	Adriatic Sea	Adriatic Sea	Black Sea	English Channel (Tamar estuary)	English Channel (Plymouth)
distribution	Celtic Sea	Antarctica, Labrador Sea, North Sea	Gulf of Mexico, New Zealand, Arabian Gulf	Caspian Sea Mediteranean	Baltic Sea (Kiel)	Mediterranen	Tyrrhenia Sea, English chanel	Adriatic Sea, North Sea, Atlantic	Adriatic Sea	Adriatic Sea	Black Sea	English Channel (Tamar estuary)	English Channel (Plymouth)
references	this study	Tillmann et al. 2022 [3]	Gomez et al. 2022 [9]	Ostenfeld 1902 [23] Schiller 1933 [31]	Lohman 1908 [27]	Schiller 1918 [32]	Puigserver & Zingone 2002 [6]	Schiller 1918 [32]	Schiller 1918 [32]	Schiller 1928 [33]	Krakhmalny & Terenko 2004 [7]	Bursa 1959 [34]	Bursa 1959 [34]

^1^ Description of both C.H. Ostenfeld (in white) for *P. cordatum* and of J. Schiller (in grey) for *P. minimum* are given. ^2^ Dodge [1] considered this species a synonym of *P. scutellum* Schröder. ^3^ Dodge [1] considered red this species a synonym of *P. nanum.* It is important to note that the taxon analysed by Dodge and Bibby [35] and Dodge [1] as *P. pusillum* today is identified as *P. nux* [6]. ^4^ Dodge [1] considered this species a synonym of *P. balticum.* ^5^ Dodge [1] considered this species a synonym of *P. minimum.* ^6^ In J. Schiller’s drawing and description, the flagella emerge from a deeply indented apical area formed by one of the thecal plates. This would make *P. ovum* distinct from all other small, roundish species of *Prorocentrum*. ^7^ A round nucleus in posterior position was reported by Berdieva et al. [36]. ^8^ Wulff (1916) [37] described the nucleus of *P. balticum* as round and located somewhat eccentrically in almost median position. ^9^ Prologue reads: “Almost always one short spine on one of the bumps”. However, no “spine” is visible in J. Schiller’s drawing. ^10^ The length of the spine is reported as 1.5–2 µm. ^11^ According to C.H. Ostenfeld’s drawing showing numerous mini-dots on the surface, these are spines not pores. ^12^ According to the original drawing from J. Pavillard reproduced by J. Schiller, but these mini-dots are spines rather than pores. ^13^ Not clear if the “subtle pores” described by J Schiller in fact are pores or are indicative of a spiny or knobby plate surface structure. ^14^ Protologue reads: “Some individuals are covered with tiny spines, others are smooth.” ^15^ Reported as 3.1 knobs per µm [7]. ^16^ Though 3–6 rod-shaped trichocysts inside the cells. ^17^ Periflagellar platelet number of *P. minimum* is reported as 8 in subsequent papers [38,39,40].

## Data Availability

Data presented in this study are in the article and supplementary material.

## Supplementary Materials

The following supporting information can be downloaded at: https://www.mdpi.com/article/10.3390/microorganisms11020271/s1, Table S1: Voucher list.
