# Supplementary material for: Morphological and Phylogenetic Characterisation of Prorocentrum spinulentum, sp. nov. (Prorocentrales, Dinophyceae), a Small Spiny Species from the North Atlantic
Source: Microorganisms. 2023 Jan 20;11(2):271. doi: 10.3390/microorganisms11020271 (PMC9967221; doi:10.3390/microorganisms11020271)
Supplement: Supplementary file 1 [file microorganisms-11-00271-s001.zip › microorganisms-2146826-supplementary.pdf]

Morphological and phylogenetic characterisation

of *Prorocentrum spinulentum*, sp. nov.

(Prorocentrales, Dinophyceae),

a small, spiny species from the North Atlantic

Urban Tillmann, Marc Gottschling, Stephan Wietkamp, Mona Hoppenrath

Supplementary Materials

Table S1. Voucher list. All names are given under the rules of the ICN, including the author standard forms [1]. Abbreviation: n. inf., no information. If 'holotype' or 'epitype' is noted for a species name, then it refers to material, from which the type was prepared.

Species name with author	Strain No.	Locality	Date	Collector(s) [isolator]	GenBankNo(s)	Reference
outgroup						
Amphisolenia bidentata Schröd. (chimeric as used in Orr et al. 2012)	LE392	Indian Ocean, off Australia (16°02'S, 119°20'E)	fall 2006	n.inf.	GU196149 (SSU), FJ808682 (LSU)	[2–3]
Barrufeta resplendens (Hulburt) H.Gu, Z.Luo & K.N.Mert.	GM17	western North Atlantic, Gulf of Mexico, off USA–LA (28°52'N, 90°29'W)	Jul 29, 2014	n.inf.	KY688183 (SSU), KT203384 (ITS), KT203382 (LSU)	[4–5]
Bispinodinium angelaceum N.Yam. & T.Horig. (holotype)	HG236	western North Pacific, off Japan: Kyūshū, Kagoshima, off Mageshima (30°41'N, 130°50'E)	May 15, 2008	R. Terada s.n.	AB762397 (SSU), AB762398 (LSU)	[6]
Dinophysis acuminata Clap. & J.Lachm.	clone 2	eastern North Atlantic, off Spain: Ría de Pontevedra (42°22'N, 8°47'W)	Jun 12, 2006	n.inf.	KF871422 (SSU), AM931580 (ITS), KF871410 (LSU)	[7–8]
Dinophysis acuminata Clap. & J.Lachm.	DARU2013	western North Pacific, Sea of Japan, off Russia: Primorsky, Vladivostok	Aug 25, 2013	n.inf.	KJ508017 (SSU), KJ508016 (ITS), KJ508015 (LSU)	Efimova et al. (unpubl. 2014)
Dinophysis caudata Kent	FTL69	western North Atlantic, off USA–FL: Ft. Lauderdale (26°05'N, 80°03'W)	Feb 26, 2008	n.inf.	EU780644 (SSU+ITS+LSU)	[9]
Gymnodiniaceae sp.	uncultivated clone W8eB2	Arctic Ocean, off Canada: Nanavut, Ellesmere Island, Ward Hunt Lake (83°05'N, 74°10'W)	Aug 15, 2008	n.inf.	JF730844 (SSU)	[10]
Gymnodinium aureolum (Hulburt) Gert Hansen	GeoB 232	Mediterranean Sea, Ionian Sea, off Italy: Gulf of Taranto (40°07'N, 17°19'E)	Oct 26, 2002	D. Saracino [M. Kirsch] s.n.	KJ481834 (SSU+ITS+LSU)	[11]

Gymnodinium aureolum (Hulburt) Gert Hansen	MUCC284	n.inf.	n.inf.	n.inf.	AF022196 (SSU)	[12]
"*Gymnodinium*" *catenatum* H.W.Graham	GnCt01	eastern Indian Ocean, East China See, off South Korea: Nanpo, Jinhae Bay	n.inf.	n.inf.	DQ785882 (rRNA)	[13]
Gymnodinium fuscum var. *rubrum* Baumeister ex Romeikat, Knechtel & Gottschling (epitype)	GeoM*864 (≡ CCAC9044B, CCAP1117/10)	Germany: Bavaria, Traunstein, Seeon, peat bog (47°59'N, 12°26'E, 536m)	Jun 28, 2017	C. Romeikat, M. Gottschling & H. Reich [C. Romeikat] D099	MK405489 (SSU+ITS+LSU)	[14]
Gymnodinium plasticum Na Wang, Z.Luo, K.N.Mert., F.M.G.McCarthy & H.Gu (holotype)	TIO826	Canada: Ontario, Plastic Lake (45°18'N, 79°23'E)	n.inf.	n.inf.	KY688188 (SSU), KY688186 (ITS), KY688184 (LSU)	[5]
"*Gymnodinium*" *smaydae* N.S.Kang, H.J.Jeong & Moestrup (holotype)	GSSW10	western North Pacific, Yellow Sea, off South Korea: Shiwha Bay (37°18′N, 126°36′E)	May, 2010	n.inf.	HG005135 (SSU+ITS+LSU)	[15]
Gymnoxanthella sp. (isolated from *Spongotrochus glacialis* Popofsky, 1908)	n.inf.	North Pacific	fall, 2010	n.inf.	AB860180 (rRNA)	[16]
Gyrodiniellum shiwhaense N.S.Kang, H.J.Jeong & Moestrup (holotype)	n.inf.	western North Pacific, Yellow Sea, off South Korea: Shiwha Bay (37°07'N, 126°08'E)	Sep 1, 2009	N.S. Kang s.n.	FR720082 (SSU+ITS+LSU)	[17]
"*Gyrodinium*" *impudicum* S.Fraga & I.Bravo	Gi-1cp	eastern Indian Ocean, East China See, off South Korea: South Jeolla, Yeosu	n.inf.	n.inf.	DQ779992 (rRNA)	[18]
Histioneis sp.	FTL62	western North Atlantic, off USA–FL: Ft. Lauderdale (26°05'N, 80°03'W)	Feb 26, 2008	n.inf.	EU780646 (SSU+ITS+LSU)	[9]
Lepidodinium viride M.Watan., S.Suda, I.Inouye, Sawaguchi & Chihara	n.inf.	South Africa	n.inf.	R.N. Pienaar s.n.	DQ499645 (SSU+ITS+LSU)	Grzebyk et al. (unpubl. 2008)
Nematodinium sp.	UBC3 (chimeric)	eastern North Pacific, off Canada: British Columbia, Vancouver Island (48°50'N, 125°08'W)	April, 2006; April 28, 2007	Anonymous BSL-2009a	FJ947038 (SSU), FJ947041 (LSU)	[19]

Nusuttodinium acidotum (Nygaard) Y.Takano & T.Horig. (reference material)	Gaci-Japan #1	Japan: Hokkaidō, Shinshinotsu, Lake Shinotsu (43°13'N, 141°39'E)	Sep 13, 2001	Y. Takano s.n.	AB921309 (rRNA)	[20]
Nusuttodinium aeruginosum (F.Stein) Y.Takano & T.Horig.	Gaer-Japan #1	Japan: Kagawa, Uto-ike Pond	Feb 9, 2001	Y. Takano s.n.	AB921311 (rRNA)	[20]
Nusuttodinium aeruginosum (F.Stein) Y.Takano & T.Horig.	Gaer-Japan #2	Japan: Hokkaidō, Akkeshi, Tokotan-numa Pond (43°00'N, 144°52'E)	Dec 7, 2001	Y. Takano s.n.	AB921312 (rRNA)	[20]
Nusuttodinium amphidinoides (Geitler) Y.Takano & T.Horig. (reference material)	Aamp-Japan #b	Japan: Hokkaidō, Sapporo (43°04'N, 141°21'W)	Apr 9, 2003	Y. Takano s.n.	AB921307 (rRNA)	[20]
Nusuttodinium desymbiontum R.Onuma, K.Watanabe & T.Horig.	Fukaura	western North Pacific, Sea of Japan, off Japan: Honshū, Aomori, Fukaura	May 5, 2012	R. Onuma s.n.	LC027036 (SSU), LC027054 (LSU)	[21]
Nusuttodinium poecilochroum (J.Larsen) Y.Takano & T.Horig.	Hokuto	western North Pacific, off Japan: Hokkaidō, Hokuto	May 12, 2012	R. Onuma s.n.	LC027047 (SSU), LC027065 (LSU)	[21]
Ornithocercus magnificus F.Stein	CBC4L7	western North Atlantic, off USA–VA: shelf break off lower Chesapeake Bay (36°20'N, 74°44'W)	Oct 15, 2007	n.inf.	EU780649 (SSU+ITS+LSU)	[9]
Prodinophysis rapa (F.Stein) Balech	CBC4L5	western North Atlantic, off USA–VA: shelf break off lower Chesapeake Bay (36°20'N, 74°44'W)	Oct 15, 2007	n.inf.	EU780655 (SSU+ITS+LSU)	[9]
Prodinophysis cf. *rotundata* (Clap. & J.Lachm.) Balech	FTL121	western North Atlantic, off USA–FL: Ft. Lauderdale (26°05'N, 80°03'W)	Feb 26, 2008	n.inf.	EU780657 (SSU+ITS+LSU)	[9]
Pseudocochlodinium profundisulcus Zhangxi Hu, N.Xu, H.Gu, M.Iwataki, K.Takahashi, Y.Z.Tang & Matsuoka	n.inf.	western North Pacific, South China Sea, off China: Zhuhai, Pearl River Estuary (22°10'N, 113°38'E)	Aug 24, 2011	n.inf.	JX967270 (SSU+ITS+LSU)	[22]
Spiniferodinium galeiforme T.Horig. & Chihara	n.inf.	n.inf.	n.inf.	M. Tamura s.n.	AB921297 (SSU), AB921298 (LSU)	[20]

Spiniferodinium limneticum (Wołosz.) Kretschmann & Gottschling	GeoM 517 (≡ CCAC5092B, CCBA AA-276)	Poland: Lesser Poland, Tatra, Zakopane (49°17'N, 19°57'E)	Sep 11, 2012	M. Gottschling, C. Zinßmeister, N.H. Filipowicz & P.M. Owsianny [J. Kretschmann P6] PL002	KR362900 (SSU+ITS+LSU)	[23]
Spiniferodinium palustre (A.J.Schill.) Kretschmann & Gottschling (reference material)	GeoM*719	Poland: Lesser Poland, Tatra, Litworowy Staw Gąsienicowy (49°14'N, 20°00'E, 1618m)	Sep 22, 2015	P.M. Owsianny, K. Trawiński & G. Marciniak [J. Kretschmann] PL018	MH497023 (SSU+ITS+LSU), MH497042 (LSUd8d10)	[24]
Spiniferodinium palustre (A.J.Schill.) Kretschmann & Gottschling	n.inf.	Japan: Hokkaidō, Lake Shikotsu	Oct 23, 2005	Y. Takano s.n.	AB921299 (SSU), AB921300 (LSU)	[20]
Warnowia sp.	BC	eastern North Pacific, off Canada: British Columbia, Vancouver Island (48°50'N, 125°08'W)	May 2, 2007	Anonymous BSL-2009a	FJ947040 (SSU), FJ947042 (LSU)	[19]
<u>Prorocentralean dinophytes</u>						
Adenoides sinensis H.Gu, Xintian Li & Z.Luo	DF386	n.inf.	n.inf.	n.inf.	OP363636 (SSU), OP369285 (ITS), OP363638 (LSU)	Guo (unpubl. 2022)
Chrysodinium ballux (N.Yamada, Dawut, R.Terada & T.Horig.) F.Gómez, Y.Nakam. & L.F.Artigas (holotype)	HG177	western North Pacific, off Japan: Kyūshū, Kagoshima, Takeshima (30°49'N, 130°24’E)	May 10, 2011	T. Horiguchi	LC054938 (SSU), LC375159 (LSU)	[25]
Plagiodinium belizeanum M.A.Faust & Balech	KW001	western North Pacific, East China Sea, off Japan: Okinawa, Itoman, Odo (26°04'N, 127°41'E)	Apr 15, 2015	n.inf.	KX008973 (SSU), KX008972 (LSU)	[26]
Plagiodinium sp.	HG225	western North Pacific, off Japan: Kyūshū, Kagoshima, Takeshima (30°49'N, 130°24’E)	May 30, 2011	T. Horiguchi	LC054937 (SSU), LC375160 (LSU)	[25]

Prorocentrum concavum Fukuyo (holotype of *Prorocentrum arabianum* S.L.Morton & M.A.Faust)	NCMA1724	western Indian Ocean, Arabian Sea, Gulf of Oman, off Oman (23°34’N, 58°52'E)	May, 1995	J. Stirn [S. Brett] OM-3	EU927555 (ITS), DQ336184 (LSU)	Ferrell & Beaton (unpubl. 2008), [28]
Prorocentrum cordatum (Ostenf.) J.D.Dodge	D127	eastern Indian Ocean, off South Korea: Tongyeong	n.inf.	n.inf.	JX402086 (rRNA)	Cheon & Ki (unpubl. 2013)
Prorocentrum cordatum (Ostenf.) J.D.Dodge	NCMA1329 (≡ CCCM541, EXUV)	western North Atlantic, off USA–NY: New York, Long Island, Great South Bay (40°40'N, 73°15'W)	Jul 1, 1958	I. Pinter s.n.	DQ336060 (SSU), EU927539 (ITS), EU532479 (LSU)	Ferrell & Beaton (unpubl. 2008), [29–30]
Prorocentrum cordatum (Ostenf.) J.D.Dodge	PIPV1	eastern North Pacific, Gulf of California, off Mexico: Baja California, Bahía de La Paz	2000	[L. Morquecho] s.n.	JQ616823 (SSU+ITS+LSU), JQ616845 (LSU)	[31]
Prorocentrum cordatum (Ostenf.) J.D.Dodge	PMDH01	western North Pacific, East China Sea, off China: Fujian	n.inf.	n.inf.	DQ028763 (SSU), DQ054538 (ITS), DQ054539 (LSU)	Hou et al. (unpubl. 2005)
Prorocentrum donghaiense D.D.Lu	NORCCA K-1260	eastern North Atlantic, off Spain: Canary Islands, La Gomera, San Sebastián	2009	[G. Hansen] s.n.	MK713637 (SSU), MK713638 (ITS), MK713639 (LSU)	[44]
Prorocentrum donghaiense D.D.Lu	LIMS-PS-2540	western North Pacific, East China Sea, Gamak Bay, off South Korea: South Jeolla	May, 2017	n.inf.	MH729037 (SSU), MK217267 (ITS), MH729045 (LSU)	[44]
Prorocentrum donghaiense D.D.Lu (chimeric)	n.inf.	western North Pacific, East China Sea	2002	S. Lin s.n.	DQ336054 (SSU), AY465116 (ITS), AY822610 (LSU)	[29, 45]
Prorocentrum cf. *emarginatum* Fukuyo	X2P3	western North Pacific, South China Sea, off China: Hainan, Sanya (18°14'N, 109°22'E)	Aug 19, 2014	n.inf.	KY010245 (ITS), KY010260 (LSU)	[34]
Prorocentrum foveolatum Croome & P.A.Tyler (reference material)	PFBL01	Australia: Tasmania, Bruny Island, Big Lagoon	Feb, 2002	[I. Pearce] s.n.	AY259173 (LSU)	[35]

Prorocentrum fukuyoi Sh.Murray & Y.Nagahama (holotype)	SM19 (≡ CS867)	western South Pacific, Tasman Sea, off Australia: NWS, Sydney, Port Botany (34°00'S, 151°14'E)	Apr, 2003	n.inf.	DQ336191 (LSU)	[28]
Prorocentrum fukuyoi Sh.Murray & Y.Nagahama	W091	South China Sea	n.inf.	n.inf.	MK547117 (SSU), MK605054 (ITS), MK605081 (LSU), MK544031 (LSUd8d10)	Yiu et al. (unpubl. 2020)
Prorocentrum gracile F.Schütt	TIO419	western North Pacific, South China Sea, Daya Bay, off China: Guangdong, Shenzhen (22°34'N, 114°36E)	Nov 11, 2016	n.inf.	ON318854 (ITS), ON318411 (LSU)	[36]
Prorocentrum gracile F.Schütt	W089	South China Sea	n.inf.	n.inf.	MK547125 (SSU), MK605061 (ITS), MG914043 (LSU), MK544040 (LSUd8d10)	Yiu et al. (unpubl. 2020)
Prorocentrum hoffmannianum M.A.Faust	NCMA683 (≡ PL200A)	western North Atlantic, off USA–FL: Knight Key (24°42'N, 81°08'W)	Nov 1, 1985	J. Bomber [J. Bomber] s.n.	KF885225 (SSU+ITS+LSU), KC622310 (SSU+ITS+LSU)	Lee (unpubl. 2013), [37]
Prorocentrum koreanum M.S.Han, S.Y.Cho & P.B.Wang	BGERL02	western North Pacific, South China Sea, Beibu Gulf, off China	n.inf.	Y. Xu s.n.	MW979822 (SSU), MW999295 (ITS), MW979767(LSU)	Xu (unpubl. 2021)
Prorocentrum koreanum M.S.Han, S.Y.Cho & P.B.Wang (holotype)	LMBEV9	eastern Indian Ocean, East China See, off South Korea: Jangmok	n.inf.	n.inf.	KP711350 (SSU), KP711351 (ITS), KP711352 (LSU)	[38]
Prorocentrum leve M.A.Faust, Kibler, Vandersea, P.A.Tester & Litaker (holotype)	NCMA2634	western North Atlantic, Caribbean Sea, off Belize: Stann Creek, Twin Cays (16°50N, 88°06'W)	May 21, 2002	S. Kibler [S. Kibler] s.n.	DQ238043 (SSU+ITS+LSU)	[39]
Prorocentrum lima (Ehrenb.) F.Stein	NCMA685 (≡ PL2V)	eastern North Atlantic, off Spain: Galicia, Ría de Vigo (42°14'N, 8°48'W)	n.inf.	I. Bravo s.n.	AB189765 (ITS), DQ336179 (LSU)	Ferrell & Beaton (unpubl. 2008), [40–41]

Prorocentrum lima (Ehrenb.) F.Stein	NCMA1370 (≡ FIT#69)	western North Atlantic, off USA–FL: Knight Key (24°42'N, 81°08'W)	n.inf.	J. Bomber s.n.	EU927507 (ITS), DQ336180 (LSU)	Ferrell & Beaton (unpubl. 2008), [40]
Prorocentrum lima (Ehrenb.) F.Stein (epitype of *Cryptomonas lima* Ehrenb. and *Exuviaella marina* Cienk.)	NCMA1743 (≡ PA)	Atlantic Ocean, off Canada: Nova Scotia, Lunenburg, Mahone Bay (44°30'N, 64°10'W)	n.inf.	[C. Brown & N. Lewis] s.n.	AB189767 (ITS)	Ferrell & Beaton (unpubl. 2008), [41]
Prorocentrum micans Ehrenb. (epitype)	A10	Baltic Sea, off Germany: Schleswig-Holstein, Kiel (54°21'N, 10°09'E)	Oct 20, 2017	K.J.S. Meier [U. Tillmann A10] s.n.	MK405477 (rRNA)	[42]
Prorocentrum micans Ehrenb.	NCMA1589 (≡ 992M3)	USA–RI: Narragansett Bay (41°36'N, 71°24'W)	Sep 19, 1992	P. Hargraves [P. Hargraves] s.n.	EU780638 (SSU+ITS+LSU)	[9]
Prorocentrum minimum (Pavill.) J.Schiller	RCC922 (≡ Biosope_182_FL1-1)	Eastern South Pacific, off Chile (33°21'S, 78°06'W)	Dec 4, 2004	[L. Garczarek & D. Marie] Bioscope STB20	FJ823585 (ITS)	[43]
Prorocentrum pervagatum Tillmann, Hoppenrath & Gottschling	CA01	Southern Ocean, Potter Cove, off UK: South Shetland Islands, King George Island (62°14'S, 58°42'W)	Jan, 2014	U. Tillmann [Dallmann 2014] [U. Tillmann] s.n.	OP094108 (SSU+ITS+LSU)	[47]
Prorocentrum pervagatum Tillmann, Hoppenrath & Gottschling	DINO:1	western South Atlantic Antarctica: Ross Sea	summer 2017	[P-ROSE]	MT831988 (ITS+LSU)	[48]
Prorocentrum pervagatum Tillmann, Hoppenrath & Gottschling (holotype)	PM01	western North Atlantic, Labrador Sea (56°50'N, 52°13'W, –10m)	Jun 28, 2017	U. Tillmann [Maria S: Merian 65] [U. Tillmann] s.n.	OP094113 (SSU+ITS+LSU)	[47]
Prorocentrum playfairii Croome & P.A.Tyler (reference material)	PPWL01	Australia: Tasmania, Windmill Lagoon	Jan, 2001	[I. Pearce] s.n.	AY259174 (LSU)	[35]
Prorocentrum redfieldii Bursa	BGERL44	western North Pacific, South China Sea, Beibu Gulf, off China	n.inf.	Y. Xu s.n.	MW979864(SSU), MW999337 (ITS), MW979809 (LSU)	Xu (unpubl. 2021)
Prorocentrum rhathymum A.R.Loebl., Sherley & R.J.Schmidt	NMN16	western North Pacific, South China Sea, off Malaysia: Sabah	n.inf.	n.inf.	FJ842096 (SSU), FJ155840 (ITS), EF566745 (LSU)	[49–50]

Prorocentrum rhathymum A.R.Loebl., Sherley & R.J.Schmidt	PRJJ1	western North Pacific, East China Sea, off Republic of South Korea: Jeju (33°52'N, 126°54'E)	Feb, 2009	n.inf.	HF565181 (SSU+ITS+LSU)	[51]
Prorocentrum rostratum F.Stein	PR1V	n.inf.	n.inf.	n.inf.	EU244471 (ITS)	Rial et al. (unpubl. 2007)
Prorocentrum shikokuense Hada	Prosh-18S-28S_Apr1	Mediterranean Sea, Adriatic Sea, off Italy: Apulia, Brandisi (40°39'N, 17°59'E)	Sep 20, 2018	n.inf.	MZ593905 (SSU+ITS+LSU)	[46]
Prorocentrum sp.	N505T_64	South China Sea	n.inf.	n.inf.	GU942241 (ITS)	Li (unpubl. 2010)
Prorocentrum sp.	NCMA1517	eastern South Pacific, off Ecuador (2°32'S, 84°12'W)	May 7, 1991	L. Polans s.n.	AY803742 (SSU), EU927560 (ITS), AY833515 (LSU)	Ferrell & Beaton (unpubl. 2008), [29, 32]
Prorocentrum sp.	RCC848	eastern South Pacific (32°24'S, 86°47'W, –20m)	Dec 1, 2004	[L. Garczarek & D. Marie] STB17	EU106736 (SSU), EU927559 (ITS)	Ferrell & Beaton (unpubl. 2008), [33]
Prorocentrum sp.	UTSPH2D4	western South Pacific, Tasman Sea, off Australia: NWS, Sydney, Port Hacking (34°07'S, 151°13'E)	Sep, 2018	n.inf.	MW024110 (SSU), MW024117 (ITS), MW024106 (LSU)	[27]
Prorocentrum spinulentum Tillmann, Gottschling & Hoppenrath (holotype)	1D3	eastern North Atlantic, Celtic Sea, off Ireland (51°01'N, 9°04'W)	Jul 26, 2018	U. Tillmann [Heincke] [U. Tillmann] 22	OQ220501 (SSU), OQ220500 (ITS+LSU)	this study
Prorocentrum cf. *spinulentum* Tillmann, Gottschling & Hoppenrath	NCMA1529	Eastern South Pacific, off Ecuador (2°40'S, 82°43'W)	Sep 8, 1992	L. Polans s.n.	EU927540 (ITS)	Ferrell & Beaton (unpubl. 2008)
Prorocentrum cf. *spinulentum* Tillmann, Gottschling & Hoppenrath	QUCCCM86	Qatar: Arabian Gulf	2013	n.inf.	KX853192 (ITS), KX853176 (LSU)	[52]

Prorocentrum cf. *spinulentum* Tillmann, Gottschling & Hoppenrath	VGO365	eastern North Atlantic, off Spain: Galicia, Ría de Vigo	Oct 10, 2010	n.inf.	EU244472 (ITS)	Rial et al. (unpubl. 2007)
Prorocentrum steidingerae F.Gómez, D.J.Qiu & Senjie Lin (holotype)	NCMA687 (≡ FIT#182, PM200A)	western North Atlantic, off USA–FL: Knight Key (24°42'N, 81°08'W)	Nov 1, 1985	J. Bomber [J. Bomber] s.n.	EU287485 (SSU), EU927554 (ITS), DQ336183 (LSU)	Ferrell & Beaton (unpubl. 2008), [28, 40]
Prorocentrum texanum Henrichs, Steid., P.S.Scott & L.Campbell (holotype)	PrTX B	USA–TX: Ship Channel, Port Aransas (27°50′N, 97°03′W)	Feb 3, 2010	D.W. Henrichs s.n.	JQ390504 (SSU), JQ390505 (ITS+LSU)	[53]
Prorocentrum thermophilum F.Gómez, Tangcheng Li, Hu.Zhang & Senjie Lin	NCMA1260 (≡ IVC3, IVC3AX)	western North Atlantic, Gulf of Mexico	Feb 1, 1981	L. Pavasoli [L. Pavasoli] s.n.	OP256708 (SSU), EU927547 (ITS), OP231463 (LSU)	Ferrell & Beaton (unpubl. 2008) [47]
Prorocentrum cf. *thermophilum* F.Gómez, Tangcheng Li, Hu.Zhang & Senjie Lin	QUCCCMSS1-13	Qatar: Arabian Gulf	2013		KX853197 (ITS), KX853186 (LSU)	[52]
Prorocentrum tsawwassenense Hoppenrath & B.S.Leander	IFR456	France: Groix Island	Jul 31, 2007	N. Chomérat NC-2013	JX912182 (LSU)	[54]
Prorocentrum tsawwassenense Hoppenrath & B.S.Leander	KW-P.twas1	Kuwait	n.inf.	W. Ismail & J. Larsen s.n.	MH669281 (LSU)	Ismail (unpubl. 2018)
Prorocentrum tsawwassenense Hoppenrath & B.S.Leander	TIO304	western North Pacific, Yellow Sea, off China: Shangdong, Qingdao (36°03'N, 120°22'E)	Oct 14, 2015	n.inf.	OP764434 (ITS), OP764421 (LSU)	[55]
Pseudadenoides kofoidii (Herdman) F.Gómez, R.Onuma, Artigas & T.Horig.	PSE6	eastern North Atlantic, off France: Hauts-de-France, Wimereux (50º46'N, 1º37'E)	Jun, 2011	n.inf.	LC002843 (SSU), LC002848 (LSU)	[56]
Pseudadenoides kofoidii (Herdman) F.Gómez, R.Onuma, Artigas & T.Horig.	NCMA1891 (≡ NEPCC683a)	eastern North Pacific, Boundary Bay, off Canada: British Columbia (49°00'N, 123°00'W)	Apr 1, 1988	D. Jacobsen [E. Simons] s.n.	KX000289 (SSU), JX262493 (ITS), KX000293 (LSU)	Yu et al. (unpubl. 2009), [57–58]

References

1. Brummitt, R.K.; Powell, C.E. *Authors of plant names: A list of authors of scientific names of plants, with recommended standard forms of their names, including abbreviations*; Royal Botanic Gardens: Kew, 1992.
2. Jensen, M.H.; Daugbjerg, N. Molecular phylogeny of selected species of the order Dinophysiales (Dinophyceae)—Testing the hypothesis of a dinophysioid radiation. *Journal of Phycology* **2009**, *45*, 1136–1152.
3. Daugbjerg, N.; Jensen, M.H.; Hansen, P.J. Using nuclear-encoded LSU and SSU rDNA sequences to identify the eukaryotic endosymbiont in *Amphisolenia bidentata* (Dinophyceae). *Protist* **2013**, *164*, 411–422.
4. Gu, H.; Luo, Z.; Mertens, K.N.; Price, A.M.; Turner, R.E.; Rabalais, N.N. Cyst-motile stage relationship, morphology, ultrastructure, and molecular phylogeny of the gymnodinioid dinoflagellate *Barrufeta resplendens* comb. nov., formerly known as *Gyrodinium resplendens*, isolated from the Gulf of Mexico. *Journal of Phycology* **2015**, *51*, 990–999.
5. Wang, N.; Luo, Z.; Mertens, K.N.; McCarthy, F.M.G.; Gu, L.; Gu, H. Cyst-motile stage relationship and molecular phylogeny of a new freshwater dinoflagellate *Gymnodinium plasticum* from Plastic Lake, Canada. *Phycological Research* **2017**, *65*, 312–321.
6. Yamada, N.; Terada, R.; Tanaka, A.; Horiguchi, T. *Bispinodinium angelaceum* gen. et sp. nov. (Dinophyceae), a new sand-dwelling dinoflagellate from the seafloor off Mageshima Island, Japan. *Journal of Phycology* **2013**, *49*, 555–569.
7. Raho, N.; Pizarro, G.; Escalera, L.; Reguera, B.; Marín, I. Morphology, toxin composition and molecular analysis of *Dinophysis ovum* Schütt, a dinoflagellate of the "*Dinophysis acuminata* complex". *Harmful Algae* **2008**, *7*, 839–848.
8. Raho, N.; Rodríguez, F.; Reguera, B.; Marín, I. Are the mitochondrial *cox1* and *cob* genes suitable markers for species of *Dinophysis* Ehrenberg? *Harmful Algae* **2013**, *28*, 64–70.
9. Handy, S.M.; Bachvaroff, T.R.; Timme, R.E.; Coats, D.W.; Kim, S.; Delwiche, C.F. Phylogeny of four Dinophysiacean genera (Dinophyceae, Dinophysiales) based on rDNA sequences from single cells and environmental samples. *Journal of Phycology* **2009**, *45*, 1163–1174.
10. Charvet, S.; Vincent, W.F.; Lovejoy, C. Chrysophytes and other protists in High Arctic lakes: Molecular gene surveys, pigment signatures and microscopy. *Polar Biology* **2012**, *35*, 733–748.
11. Tillmann, U.; Gottschling, M.; Nézan, E.; Krock, B.; Bilien, G. Morphological and molecular characterization of three new *Azadinium* species (Amphidomataceae, Dinophyceae) from the Irminger Sea. *Protist* **2014**, *165*, 417–444.
12. Saunders, G.W.; Hill, D.R.A.; Sexton, J.P.; Andersen, R.A. Small-subunit ribosomal RNA sequences from selected dinoflagellates: Testing classic evolutionary hypotheses with molecular systematic methods. *Plant Systematics and Evolution (Supplement)* **1997**, *11*, 237–259.
13. Ki, J.-S.; Han, M.-S. Informative characteristics of 12 divergent domains in complete large subunit rDNA sequences from the harmful dinoflagellate genus, *Alexandrium* (Dinophyceae). *Journal of Eukaryotic Microbiology* **2007**, *54*, 210–219.
14. Romeikat, C.; Knechtel, J.; Gottschling, M. Clarifying the taxonomy of *Gymnodinium fuscum* var. *rubrum* from Bavaria (Germany) and placing it in a molecular phylogeny of the Gymnodiniaceae (Dinophyceae). *Systematics and Biodiversity* **2020**, *18*, 102–115.
15. Kang, N.S.; Jeong, H.J.; Moestrup, Ø.; Lee, S.Y.; Lim, A.S.; Jang, T.Y.; Lee, K.H.; Lee, M.J.; Jang, S.H.; Potvin, É., et al. *Gymnodinium smaydae* n. sp., a new planktonic phototrophic dinoflagellate from the coastal waters of Western Korea: Morphology and molecular characterization. *Journal of Eukaryotic Microbiology* **2014**, *61*, 182–203.

16. Ishitani, Y.; Ujiie, Y.; Takishita, K. Uncovering sibling species in Radiolaria: Evidence for ecological partitioning in a marine planktonic protist. *Molecular Phylogenetics and Evolution* **2014**, *78*, 215–222.
17. Kang, N.S.; Jeong, H.J.; Moestrup, Ø.; Park, T.G. *Gyrodiniellum shiwhaense* n. gen., n. sp., a new planktonic heterotrophic dinoflagellate from the coastal waters of Western Korea: Morphology and ribosomal DNA gene sequence. *Journal of Eukaryotic Microbiology* **2011**, *58*, 284–309.
18. Ki, J.-S.; Han, M.-S. Cryptic long internal repeat sequences in the ribosomal DNA ITS1 gene of the dinoflagellate *Cochlodinium polykrikoides* (Dinophyceae): A 101 nucleotide six-repeat track with a palindrome-like structure. *Genes & Genetic Systems* **2007**, *82*, 161–166.
19. Hoppenrath, M.; Bachvaroff, T.R.; Handy, S.M.; Delwiche, C.F.; Leander, B.S. Molecular phylogeny of ocelloid-bearing dinoflagellates (Warnowiaceae) as inferred from SSU and LSU rDNA sequences. *BMC Evolutionary Biology* **2009**, *9*, 116.
20. Takano, Y.; Yamaguchi, H.; Inouye, I.; Moestrup, Ø.; Horiguchi, T. Phylogeny of five species of *Nusuttodinium* gen. nov. (Dinophyceae), a genus of unarmoured kleptoplastidic dinoflagellates. *Protist* **2014**, *165*, 759–778.
21. Onuma, R.; Watanabe, K.; Horiguchi, T. *Pellucidodinium psammophilum* gen. & sp. nov. and *Nusuttodinium desymbiontum* sp. nov. (Dinophyceae), two novel heterotrophs closely related to kleptochloroplastidic dinoflagellates. *Phycologia* **2015**, *54*, 192–209.
22. Qiu, D.; Huang, L.; Liu, S.; Zhang, H.; Lin, S. Apical groove type and molecular phylogeny suggests reclassification of *Cochlodinium geminatum* as *Polykrikos geminatum*. *PLoS One* **2013**, *8*, e71346.
23. Kretschmann, J.; Filipowicz, N.H.; Owsianny, P.M.; Zinßmeister, C.; Gottschling, M. Taxonomic clarification of the unusual dinophyte *Gymnodinium limneticum* Wołosz. (Gymnodiniaceae) from the Tatra Mountains. *Protist* **2015**, *166*, 621–637.
24. Žerdoner Čalasan, A.; Kretschmann, J.; Gottschling, M. They are young, and they are many: Dating freshwater lineages in unicellular dinophytes. *Environmental Microbiology* **2019**, *21*, 4125–4135.
25. Yamada, N.; Dawut, M.; Terada, R.; Horiguchi, T. *Plagiodinium ballux* sp. nov. (Dinophyceae), a deep (36 m) sand dwelling dinoflagellate from subtropical Japan. *Phycological Research* **2019**, *67*, 12–20.
26. Wakeman, K.C.; Hoppenrath, M.; Yamaguchi, A.; Gavelis, G.S.; Leander, B.S.; Nozaki, H. Ultrastructure of the marine benthic dinoflagellate *Plagiodinium belizeanum* (Dinophyceae) from the southeast Pacific island of Okinawa, Japan. *Phycologia* **2018**, *57*, 209–222.
27. Larsson, M.E.; Bramucci, A.R.; Collins, S.; Hallegraeff, G.; Kahlke, T.; Raina, J.B.; Seymour, J.R.; Doblin, M.A. Mucospheres produced by a mixotrophic protist impact ocean carbon cycling. *Nature Communications* **2022**, *13*, 1301.
28. Murray, S.A.; Nagahama, Y.; Fukuyo, Y. Phylogenetic study of benthic, spine-bearing prorocentroids, including *Prorocentrum fukuyoi* sp. nov. *Phycological Research* **2007**, *55*, 91–102.
29. Lin, S.; Zhang, H.; Jiao, N.Z. Potential utility of mitochondrial cytochrome b and ITS mRNA editing in resolving closely related dinoflagellates: A case study of *Prorocentrum* (Dinophyceae). *Journal of Phycology* **2006**, *42*, 646–654.
30. Howard, M.D.A.; Smith, G.J.; Kudela, R.M. Phylogenetic relationships of yessotoxin-producing dinoflagellates, based on the large subunit and Internal Transcribed Spacer ribosomal DNA domains. *Applied and Environmental Microbiology* **2009**, *75*, 54–63.
31. Herrera Sepúlveda, A.; Hernandez-Saavedra, N.Y.; Medlin, L.K.; West, N. Capillary electrophoresis finger print technique (CE-SSCP): An alternative tool for the monitoring

activities of HAB species in Baja California Sur Costal. *Environmental Science and Pollution Research* **2013**, *20*, 6863–6871.

32. Chen, J.; Zhen, Y.; Mi, T.; Yu, Z. Detection of *Prorocentrum donghaiense* using sandwich hybridization integrated with nuclease protection assay. *Acta Oceanologica Sinica* **2009**, *28*, 121–126.

33. Gall, F.L.; Rigaut-Jalabert, F.; Marie, D.; Garczarek, L.; Viprey, M.; Gobet, A.; Vaulot, D. Picoplankton diversity in the South-East Pacific Ocean from cultures. *Biogeosciences* **2008**, *5*, 203–214.

34. Luo, Z.; Hua, Z.; Krock, B.; Lu, S.; Yang, W.; Gu, H. Morphology, molecular phylogeny and okadaic acid production of epibenthic *Prorocentrum* (Dinophyceae) species from the northern South China Sea. *Algal Research* **2017**, *22*, 14–30.

35. Pearce, I.; Hallegraeff, G.M. Genetic affinities, ecophysiology and toxicity of *Prorocentrum playfairii* and *P. foveolata* (Dinophyceae) from Tasmanian freshwaters. *Phycologia* **2004**, *43*, 271–281.

36. Pei, L.L.; Hu, W.J.; Wang, P.B.; Kang, J.H.; Mohame, H.F.; Wang, C.Y.; Liu, L.M.; Luo, Z.H. Morphologic and phylogenic characterization of two bloom-forming planktonic *Prorocentrum* (Dinophyceae) species and their potential distribution in the China Sea. *Algal Research* **2022**, *66*, 102788.

37. Herrera Sepúlveda, A.; Medlin, L.K.; Murugan, G.; Sierra-Beltrán, A.P.; Cruz-Villacorta, A.A.; Hernández-Saavedra, N.Y. Are *Prorocentrum hoffmannianum* and *Prorocentrum belizeanum* (Dinophyceae, Prorocentrales), the same species? An integration of morphological and molecular data. *Journal of Phycology* **2015**, *51*, 173–188.

38. Han, M.-S.; Wang, P.; Kim, J.H.; Cho, S.-Y.; Park, B.S.; Kim, J.-H.; Katano, T.; Kim, B.-H. Morphological and molecular phylogenetic position of *Prorocentrum micans* sensu stricto and description of *Prorocentrum koreanum* sp. nov. from southern coastal waters in Korea and Japan. *Protist* **2016**, *167*, 32–50.

39. Faust, M.A.; Vandersea, M.W.; Kibler, S.R.; Tester, P.A.; Litaker, R.W. *Prorocentrum levis*, a new benthic species (dinophyceae) from a mangrove island, Twin Cays, Belize. *Journal of Phycology* **2008**, *44*, 232–240.

40. Murray, S.A.; Ip, C.L.C.; Moore, R.; Nagahama, Y.; Fukuyo, Y. Are prorocentroid dinoflagellates monophyletic? A study of 25 species based on nuclear and mitochondrial genes. *Protist* **2009**, *160*, 245–264.

41. Nagahama, Y.; Murray, S.A.; Tomaru, A.; Fukuyo, Y. Species boundaries in the toxic dinoflagellate *Prorocentrum lima* (Dinophyceae, Prorocentrales), based on morphological and phylogenetic characters. *Journal of Phycology* **2011**, *47*, 178–189.

42. Tillmann, U.; Hoppenrath, M.; Gottschling, M. Reliable determination of *Prorocentrum micans* Ehrenb. (Prorocentrales, Dinophyceae) based on newly collected material from the type locality. *European Journal of Phycology* **2019**, *54*, 417–431.

43. Stern, R.F.; Andersen, R.A.; Jameson, I.; Küpper, F.C.; Coffroth, M.-A.; Vaulot, D.; Le Gall, F.; Véron, B.; Brand, J.J.; Skelton, H., et al. Evaluating the ribosomal Internal Transcribed Spacer (ITS) as a candidate dinoflagellate barcode marker. *PLoS One* **2012**, *7*, e42780.

44. Shin, H.H.; Li, Z.; Mertens, K.N.; Seo, M.H.; Gu, H.; Lim, W.A.; Yoon, Y.H.; Soh, H.Y.; Matsuoka, K. *Prorocentrum shikokuense* Hada and *P. donghaiense* Lu are junior synonyms of *P. obtusidens* Schiller, but not of *P. dentatum* Stein (Prorocentrales, Dinophyceae). *Harmful Algae* **2019**, *89*, 101686.

45. Zhang, B.-Y.; Wang, G.-C.; Zhang, Y.; Han, X.-T.; Lo, S.-H.; Qi, Y.-Z.; Zou, J.-Z.; Zeng, C.-K. 东海原甲藻 (*Prorocentrum donghaiense*) 和海洋原甲藻 APBM (*P.micans* APBM) 的 5.8S rDNA 及其转录间隔区 (ITS) 的克隆和序列分析. *Oceanologia et limnologia sinica* **2004**, *35*, 265–272.

46. Gómez, F.; Zhang, H.; Roselli, L.; Lin, S. Detection of *Prorocentrum shikokuense* in the Mediterranean Sea and evidence that *P. dentatum*, *P. obtusidens* and *P. shikokuense* are three different species (Prorocentrales, Dinophyceae). *Acta Protozoologica* **2021**, 47–59.
47. Tillmann, U.; Wietkamp, S.; Gottschling, M.; Hoppenrath, M. *Prorocentrum pervagatum* sp. nov. (Prorocentrales, Dinophyceae): A new, small, planktonic species with a global distribution. *Phycological Research* **in press**.
48. Bolinesi, F.; Saggiomo, M.; Aceto, S.; Cordone, A.; Serino, E.; Valoroso, M.C.; Mangoni, O. On the relationship between a novel *Prorocentrum* sp. and colonial *Phaeocystis antarctic*a under iron and vitamin B-12 limitation: Ecological implications for Antarctic waters. *Applied Sciences* **2020**, *10*, 6965.
49. Mohammad-Noor, N.; Moestrup, Ø.; Daugbjerg, N. Light, electron microscopy and DNA sequences of the dinoflageflate *Prorocentrum concavum* (syn. P*. arabianum*) with special emphasis on the periflagellar area. *Phycologia* **2007**, *46*, 549–564.
50. Caillaud, A.; de la Iglesia, P.; Campas, M.; Elandaloussi, L.; Fernandez, M.; Mohammad-Noor, N.; Andree, K.; Diogene, J. Evidence of okadaic acid production in a cultured strain of the marine dinoflagellate *Prorocentrum rhathymum* from Malaysia. *Toxicon* **2010**, *55*, 633–637.
51. Lim, A.S.; Jeong, H.J.; Jang, T.Y.; Kang, N.S.; Lee, S.Y.; Yoo, Y.D.; Kim, H.S. Morphology and molecular characterization of the epiphytic dinoflagellate *Prorocentrum* cf. *rhathymum* in temperate waters off Jeju Island, Korea. *Ocean Science Journal* **2013**, *48*, 1–17.
52. Al Muftah, A.; Selwood, A.I.; Foss, A.J.; Al-Jabri, H.M.; Potts, M.; Yilmaz, M. Algal toxins and producers in the marine waters of Qatar, Arabian Gulf. *Toxicon* **2016**, *122*, 54–66.
53. Henrichs, D.W.; Scott, P.S.; Steidinger, K.A.; Errera, R.M.; Abraham, A.; Campbell, L. Morphology and phylogeny of *Prorocentrum texanum* sp. nov. (Dinophyceae): A new toxic dinoflagellate from the Gulf of Mexico coastal waters exhibiting two distinct morphologies. *Journal of Phycology* **2013**, *49*, 143–155.
54. Hoppenrath, M.; Chomérat, N.; Horiguchi, T.; Schweikert, M.; Nagahama, Y.; Murray, S.A. Taxonomy and phylogeny of the benthic *Prorocentrum* species (Dinophyceae)—A proposal and review. *Harmful Algae* **2013**, *27*, 1–28.
55. Wu, Y.X.; Huang, S.N.; Krock, B.; Leaw, C.P.; Teng, S.T.; Piumsomboon, A.; Punnarak, P.; Roeroe, K.A.; Wang, N.; Gu, H.F. Cryptic speciation of benthic *Prorocentrum* (Dinophyceae) species and their potential as ecological indicators. *Journal of Sea Research* **2022**, *190*, 102304.
56. Gómez, F.; Onuma, R.; Artigas, L.F.; Horiguchi, T. A new definition of *Adenoides eludens*, an unusual marine sand-dwelling dinoflagellate without cingulum, and *Pseudadenoides kofoidii* gen. & comb. nov for the species formerly known as *Adenoides eludens*. *European Journal of Phycology* **2015**, *50*, 125–138.
57. Orr, R.J.S.; Murray, S.A.; Stüken, A.; Rhodes, L.; Jakobsen, K.S. When naked became armored: An eight-gene phylogeny reveals monophyletic origin of theca in dinoflagellates. *PLoS One* **2012**, *7*, e50004.
58. Hoppenrath, M.; Yubuki, N.; Stern, R.; Leander, B.S. Ultrastructure and molecular phylogenetic position of a new marine sand-dwelling dinoflagellate from British Columbia, Canada: *Pseudadenoides polypyrenoides* sp. nov. (Dinophyceae). *European Journal of Phycology* **2017**, *52*, 208–224.
